# A novel signature based on necroptosis-related long non-coding RNAs for predicting prognosis of patients with glioma

**DOI:** 10.3389/fonc.2022.940220

**Published:** 2022-08-10

**Authors:** Pengfei Xia, Yimin Huang, Gang Chen

**Affiliations:** ^1^ Department of Neurosurgery, The First Affiliated Hospital of Soochow University, Suzhou, China; ^2^ Department of Neurosurgery, Tongji Hospital of Tongji Medical College of Huazhong University of Science and Technology, Wuhan, China; ^3^ Department of Neurosurgery & Brain and Nerve Research Laboratory, The First Affiliated Hospital of Soochow University, Suzhou, China

**Keywords:** glioma, necroptosis, lncRNAs, prognostic, risk score

## Abstract

Necroptosis is closely related to the occurrence and development of tumors, including glioma. A growing number of studies indicate that targeting necroptosis could be an effective treatment strategy against cancer. Long non-coding RNA (lncRNA) is also believed to play a pivotal role in tumor epigenetics. Therefore, it is necessary to identify the functions of necroptosis-related lncRNAs in glioma. In this study, the transcriptome and clinical characteristic data of glioma patients from The Cancer Genome Atlas (TCGA) and Chinese Glioma Genome Atlas (CGGA) databases were collected, and the differentially expressed necroptosis-related lncRNAs in TCGA that have an impact on overall survival (OS) were screened out to construct risk score (RS) formula, which was verified in CGGA. A nomogram was constructed to predict the prognosis of glioma patients based on clinical characteristics and RS. In addition, Gene Set Enrichment Analysis (GSEA) was used to analyze the main enrichment functions of these necroptosis-related lncRNAs and the immune microenvironment. A total of nine necroptosis-related lncRNAs have been identified to construct the RS formula, and the Kaplan–Meier (K-M) survival analysis showed significantly poorer outcomes in the high RS group in both TCGA and CGGA databases. Moreover, the receiver operating characteristic (ROC) curve shows that our prediction RS model has good predictability. Regarding the analysis of the immune microenvironment, significant differences were observed in immune function and immune checkpoint between the high RS group and the low RS group. In conclusion, we constructed a necroptosis-related lncRNA RS model that can effectively predict the prognosis of glioma patients and provided the theoretical basis and the potential therapeutic targets for immunotherapy against gliomas.

## Introduction

Glioma refers to tumors that originate from brain glial cells and are the most common primary intracranial tumors in adults, accounting for 78% of all malignancies in the brain (1). According to the World Health Organization (WHO) classification, gliomas can be classified into grades I–IV, ranging from grades I and II as low-grade gliomas (LGGs) to grades III and IV as high-grade gliomas (HGGs) (2). Among them, the 5-year survival rate of LGG ranged from 30% to 70% (3), while the median total survival time for HGG was merely 15 months (4). Current therapeutic strategies include surgical resection, combined with radiotherapy, chemotherapy, and other comprehensive treatment methods. For the past decades, despite enormous investigation of glioma pathophysiology, the first-line treatment of glioma remains the combination of surgical resection followed by radiotherapy and temozolomide. There is a very limited improvement in glioma patients’ prognosis. However, the rise of immunotherapy including vaccination, blocking of immune checkpoints, oncolytic viruses, and adoptive immunotherapy using chimeric antigen receptor T cells (CAR-T) is bringing new hope to glioma patients. With the development of molecular biology, an increasing number of molecular markers have been illustrated to be of great significance for the individualized treatment and clinical prognosis of glioma, such as isocitrate dehydrogenase (IDH) mutation (5), chromosomal 1p/19q combined deletion state (6), and methylation of the promoter region of *O* ^6^-methylguanine-DNA methyltransferase (MGMT) (7). Hence, identifying more potential molecular targets could be beneficial for glioma patients.

Necroptosis is a pathway indicating programmed lysis cell death or inflammatory cell death, which plays a crucial role in killing pathogen-infected and/or damaged cells during certain degenerative or inflammatory diseases. The discovery of necrotic apoptosis suggests that cells could die in a programmed manner (8). Necroptosis can be induced by a variety of innate immune signaling pathways, including by stimulating RIG-I-like receptors, toll-like receptors (TLRs), and death receptors (9) (10) (11). These signaling pathways lead to activation of the necrotic kinase RIPK3 as well as RIPK1 (11). More specifically, RIPK3 activates the pseudokinase MLKL through phosphorylation, resulting in conformational changes and activation. The activated MLKL trans-locates onto the plasma membrane and causes changes in membrane permeability (12). Previous studies have proved that necroptosis signaling pathways are involved in the progression and prognosis of gliomas (13) (14) (15).

Long non-coding RNAs (lncRNAs) refer to RNA with a length of more than 200 nt, which does not encode protein itself but forms multilevel regulatory gene expression in the form of RNA (16). LncRNA has been reported to affect various phenotypes in gliomas. For example, LncRNA BCYRN1 inhibits glioma tumorigenesis by PTEN/AKT/p21 pathway (17), and p53-targeted lncRNA ST7-AS1 interacting with PTBP1 acts as a glioma suppressor (18). Moreover, it has been reported that necroptosis-related lncRNAs play important role in tumors. For instance, lncRNA H19-derived microRNA-675 could promote liver necroptosis (19). There are few studies focusing on the effect of necroptosis-related lncRNAs on gliomas. Therefore, clarifying the functions of necroptosis-related lncRNAs could provide a better understanding with regard to the role of necroptosis-associated lncRNA in immunotherapy and targeted therapy of glioma.

In this study, we identified and analyzed necroptosis-related lncRNAs based on The Cancer Genome Atlas (TCGA) glioma database, in which the glioma patients were diagnosed by pathological specialists according to the description of TCGA. We constructed a necroptosis-related lncRNAs formula that can predict the prognosis of glioma patients. These lncRNA signatures could be potential targets for attenuating the progression of glioma and improving the prognosis of patients.

## Materials and methods

### Datasets and clinical data

The RNA-sequencing profile and matching clinical information of glioma patients were downloaded from TCGA dataset (https://portal.gdc.cancer.gov/repository/, up to 20 March 2022); the normalized fragments per kilobase million (FPKM) format of the RNA-seq data was used to combine into a microarray dataset based on Perl program (version Strawberry-Perl-5.32.1.1, https://www.perl.org/). To validate our analysis, RNA-seq data and clinical information of glioma patients from the Chinese Glioma Genome Atlas (CGGA) database (http://www.cgga.org.cn/, up to March 2022) were also downloaded as validation groups. A total of 52 necroptosis-related genes (**Supplementary Table 1**) were acquired from Gene Set Enrichment Analysis (GSEA) (https://www.gsea-msigdb.org/gsea/) and Kyoto Encyclopedia of Genes and Genomes (KEGG) (https://www.kegg.jp/kegg/).

### Identification of necroptosis-related long non-coding RNAs

LncRNAs in TCGA and Chinese Glioma Genome Atlas (CGGA) datasets were screened by the Ensembl database (https://www.ensembl.org/), and the online Venn diagram tool (https://bioinformatics.psb.ugent.be/) was used to cross the screened lncRNAs in these two datasets and extract the common lncRNAs for further analysis. Next, the co-expression network analysis of the selected common LncRNAs and 52 known necroptosis-related genes was performed using the ‘limma’ package in R language software (version R 4.1.3, https://www.r-project.org/) to screen out the necroptosis-related lncRNAs; the filtering criterion was |correlation coefficient| <0.4 and p-value >0.001.

### Establishment and validation of a necroptosis-related long non-coding RNA prognostic model

First, necroptosis-related lncRNAs in normal brain tissue and glioma were analyzed; after screening the differentially expressed necroptosis-related lncRNAs, Cox regression analysis was used to screen the genes related to survival, and the top 10 necroptosis-related lncRNAs with the most significant survival differences were selected for further analysis. The least absolute shrinkage and selection operator (LASSO) analysis was performed to build a prognostic model; in order to avoid over-fitting, we adopted the ‘glmnet’ R package for analysis and set the coefficient parameter to 0.009. Each necroptosis-related LncRNA had an independent prognostic coefficient. Finally, the necroptosis-related lncRNAs were included in the construction of the risk score (RS). The RS formula is as follows:


$$\text{Risk score}\left(RS\right)=\sum_{i=1}^{N}\left(Expi*Coei\right)$$

*Expi* is the expression of each lncRNA. *Coei* is the prognostic coefficient of each lncRNA.

Median RS was used to define glioma patients as high risk or low risk, and Kaplan–Meier (K-M) curves were used to analyze the overall survival (OS) of glioma patients. In order to validate the probability of our prognostic necroptosis-related lncRNA RS formula, we used the same gene coefficient and RS calculation method in the CGGA dataset and analyzed and observed the survival prediction of glioma patients.

### Assessment of the necroptosis-related long non-coding RNA signature

To assess the survival prognosis of glioma patients in relation to clinical data, pathological grades, and the RS we constructed, univariate and multivariate Cox regression analyses were used based on the R package ‘survival’. The clinical data, pathological grades, and established RS were assessed for predictive accuracy using the ‘survivalROC’ package in the R software and visualized in the form of receiver operating characteristic (ROC) curves. Patients with glioma were divided into the LGG group and HGG group according to their pathological grades in TCGA database, and then the two groups were divided into the high RS group and low RS group according to the constructed median of RS, respectively. K-M has been used to analyze the prognostic prediction of the survival of patients with different pathological grades by RS. To establish clinicopathological parameters that predict survival effectiveness in patients with glioma, we developed a nomogram to predict the probability of OS at 1, 3, and 5 years in TCGA.

### Gene set and functional enrichment analyses of necroptosis-related long non-coding RNAs

Glioma patients in TCGA were stratified into the high RS group and low RS group according to the median RS. Based on the prognostic necroptosis-related lncRNAs, the GSEA was used to analyze the differences between the glioma patients in the high RS group and low RS group. Gene Ontology (GO) and KEGG were performed to explore the biological processes and signaling pathways associated with the necroptosis-related lncRNAs, the functional enrichment ‘clusterProfiler’ R package was used to perform gene annotation enrichment analysis, and the threshold was set at p < 0.05, |NES| >1, and false discovery rate (FDR) q <0.25.

### Immune cell infiltration subtypes and immune function were analyzed for necroptosis-related long non-coding RNAs

According to immune cell infiltration, which plays a vital role in gliomas, the differences in immune cells and main immune functions between the high-risk group and the low-risk group were analyzed and visualized by single-sample GSEA (ssGSEA). In addition, we performed a cell subtype analysis of immune infiltration for glioma patients based on the constructed RS combined with XCELL, TIMER, QUANTISEQ, MCPCOUNTER, EPIC, CIBERSOFT ABS, and CIBERSOFT to show the correlation between two groups. The immune cells consist of the following categories: B cells naive, B cells memory, Plasma cells, T cells CD8, T cells CD4 naive, T cells CD4 memory resting, T cells CD4 memory activated, T cells follicular helper, T cells regulatory (Tregs), T cells gamma delta, Natural killer (NK) cells resting, NK cells activated, Monocytes, Macrophages M0, Macrophages M1, Macrophages M2, Dendritic cells resting, Dendritic cells activated, Mast cells resting, Mast cells activated, Eosinophils, and Neutrophils. The threshold was set at p < 0.05.

### Investigation of the stromal, immune scores, and immune checkpoints in the cancer genome atlas

The ESTIMATE algorithm was applied to calculate the stromal scores and immune scores and combine these two scores to calculate the ESTIMATE scores; the differences between the high and low groups were analyzed. Furthermore, in order to provide therapeutic targets for clinical treatment, we also analyzed differences in immune checkpoints between the high and low RS groups.

## Results

### Acquisition of glioma expression data and identification of necroptosis-related long non-coding RNAs

**Figure 1:**
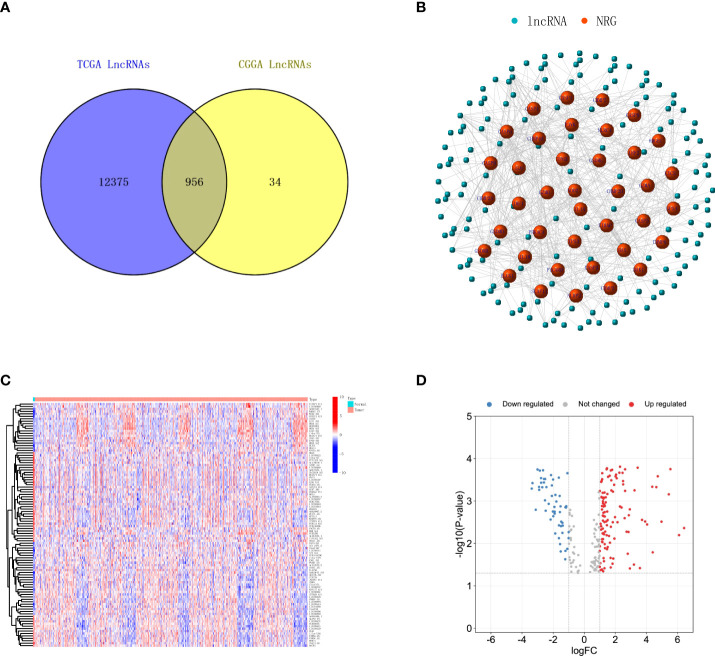
Identification of necroptosis-related lncRNAs in glioma. **(A)** Venn diagram shows the lncRNAs common to TCGA and CGGA datasets. **(B)** A co‐expression network of the necroptosis-related lncRNAs was constructed and visualized to indicate the interactions among the necroptosis-related mRNAs and lncRNAs. **(C)** Heatmap illustrates the upregulation and downregulation top 50 necroptosis-related lncRNAs with the most significant differences. **(D)** Volcano plot depicts the necroptosis-related lncRNAs. The red dots indicate upregulated lncRNAs, while blue dots indicate downregulated lncRNAs. lncRNAs, long non-coding RNAs; TCGA, The Cancer Genome Atlas; CGGA, Chinese Glioma Genome Atlas.

To establish the risk model, a TCGA glioma database with a total of five normal brain tissue samples and 698 glioma tissue samples were included. The RNA-seq expression profiles were downloaded and matched to the corresponding patients. Clinical data and RNA expression files of 1,018 glioma patients were downloaded from the CGGA database as a validation group. First, we discriminated mRNA and lncRNA in TCGA and CGGA according to genetic details in the Ensembl database (GRCh38. p13, http://asia.ensembl.org/index.html). A total of 13,331 lncRNA were found in TCGA glioma patients, 990 lncRNAs were found in the CGGA database, the Venn diagram was used to merge and take intersection, and 956 lncRNAs were found in common as shown in **Figure 1A**. Next, 52 necroptosis-related genes were retrieved from GSEA and KEGG using the ‘necroptosis’ functional term, which were used for co-expression network analysis with the common lncRNAs. The necroptosis process-related lncRNA signatures are listed in **Supplementary Table 2**. As shown in **Figure 1B**, the red dots are necroptosis-related lncRNA signatures, the blue dots are the lncRNAs consistent with |correlation coefficient| <0.4 and p-value >0.001, and the more links between them, the stronger the co-expression.

### Screening of a prognostic necroptosis-related long non-coding RNA signature

We first analyzed the screened necroptosis-related lncRNAs in TCGA database for the glioma group and normal brain tissue group and calculated the differentially expressed genes; the results showed that there was a total of 173 differentially expressed lncRNAs (**Supplementary Table 2**). We visualized the top 50 lncRNAs with the significant difference in upregulation and downregulation through heatmap, as shown in **Figure 1C**. The distribution of gene expression differences between the two groups of samples is shown by a volcanic plot, as shown in **Figure 1D**. Next, we selected 10 necroptosis-related lncRNAs with the most significant differences in overall survival as our risk score establishment. First, we deleted normal tissue samples from TCGA database.

**Figure 2:**
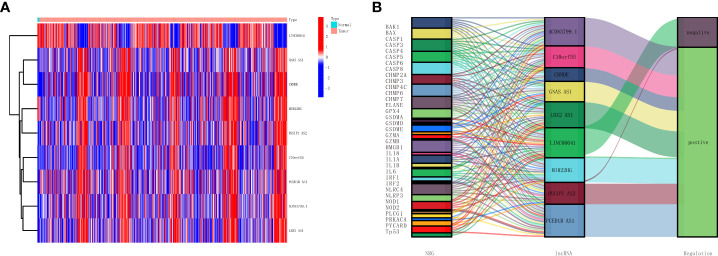
Establishment of a necroptosis-related lncRNAs prognostic model. **(A)** Heatmap presents the nine necroptosis-related lncRNAs with prognostic value in TCGA. **(B)** Sankey diagrams show the necroptosis-related mRNAs and nine necroptosis-related lncRNAs with prognostic values in TCGA. lncRNAs, long non-coding RNAs; TCGA, The Cancer Genome Atlas.

Next, to improve the accuracy of the study, glioma patients whose survival days were less than 30 days were also excluded. Cox regression was used for the rest of the glioma samples, and the selected 10 lncRNAs from the 173 differentially expressed genes lncRNAs show a top significant difference in overall survival of patients. Furthermore, we used the LASSO regression analysis to calculate the 10 lncRNAs’ survival coefficient and ruled out the uniformity lncRNAs; the results showed that a total of nine necroptosis-related lncRNAs (MIR22HG, AC083799.1, PAXIP1.AS2, C10orf55, GNAS.AS1, CRNDE, PCED1B.AS1, LBX2.AS1, and LINC00641) can influence overall survival for glioma patients independently, and the expression of these nine lncRNAs is shown in **Figure 2A**. In addition, we also conducted a co-expression network analysis of these nine lncRNAs and necroptosis-related lncRNAs, and the results are shown in the Sankey diagram in **Figure 2B**.

### Establishment and validation of a necroptosis-related long non-coding RNA prognostic model

**Figure 3:**
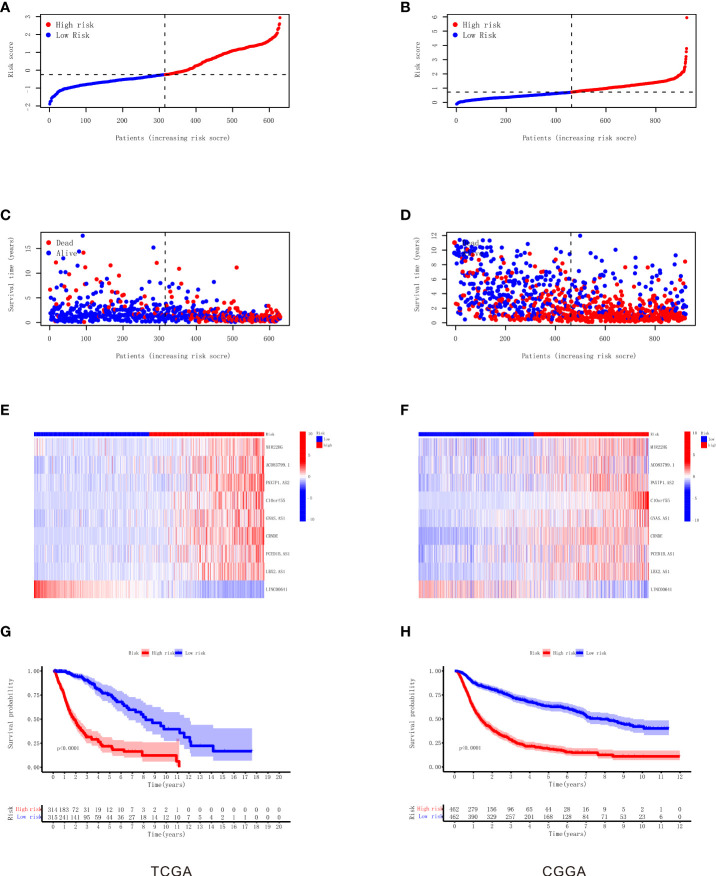
Construction and validation of the nine necroptosis-related lncRNA signatures for survival prediction. **(A)** The distribution of each sample based on the risk score in TCGA. **(B)** The distribution of each sample based on the risk score in CGGA. **(C)** The scatterplot based on the survival time and status of each sample in TCGA. The red and blue dots represent dead and alive, respectively. **(D)** The scatterplot based on the survival time and status of each sample in CGGA. The red and blue dots represent dead and alive, respectively. **(E)** The heatmap of nine necroptosis-related lncRNAs’ expression levels in the high‐risk and low‐risk groups in TCGA. **(F)** The heatmap of nine necroptosis-related lncRNAs’ expression levels in the high‐risk and low‐risk groups in CGGA. **(G)** Kaplan–Meier survival analysis of the high‐risk and low‐risk groups based on the nine necroptosis-related lncRNAs in TCGA. **(H)** Kaplan–Meier survival analysis of the high‐risk and low‐risk groups based on the nine necroptosis-related lncRNAs in CGGA. lncRNA, long non-coding RNA; TCGA, The Cancer Genome Atlas; CGGA, Chinese Glioma Genome Atlas.

As shown in **Table 1**, the correlation coefficient of nine necroptosis-related lncRNAs was calculated by LASSO regression in TCGA. We used the RS formula to calculate each patient’s score and divided the patients into high-risk and low-risk groups according to the median, as shown in **Figure 3A**, and from our results, it can be found that the mortality rate of patients in the high RS group was obviously higher than that of the group of patients with low RS, as shown in **Figure 3C**. Meanwhile, we also visualized the expression levels of nine lncRNAs in the high- and low-risk groups, as shown in **Figure 3E**. The K-M curve also shows that the high RS group had a poor overall survival rate, as shown in **Figure 3G**.

**Table 1:** Nine necroptosis-related lncRNAs in the prognostic classifier associated with overall survival in TCGA.

ID	uniCox				LASSO
	HR	HR.95L	HR.95H	p-Value	Coef
MIR22HG	1.120	1.095	1.145	<0.001	0.016
AC083799.1	1.330	1.258	1.406	<0.001	0.004
PAXIP1-AS2	1.386	1.318	1.457	<0.001	0.158
C10orf55	5.809	4.061	8.310	<0.001	0.605
GNAS-AS1	71.614	30.773	166.658	<0.001	0.416
CRNDE	1.208	1.171	1.246	<0.001	0.069
PCED1B-AS1	1.174	1.135	1.215	<0.001	0.024
LBX2-AS1	1.691	1.541	1.855	<0.001	0.043
LINC00641	0.838	0.813	0.863	<0.001	−0.070

lncRNAs, long non-coding RNAs; TCGA, The Cancer Genome Atlas; HR, hazard ratio; LASSO, least absolute shrinkage and selection operator.

To verify our RS model, we used the same gene coefficients in the CGGA database to calculate the RS of each patient according to the same formula, and we divided the patients into two groups of high and low risk according to the median, as shown in **Figure 3B**. The analysis proved that similar to TCGA results, the majority of mortality exhibit clustering in high RS, as shown in **Figure 3D**, and the expression of nine necroptosis-related lncRNAs in CGGA visualization between the high-risk and low-risk groups as shown in **Figure 3F**. The K-M survival analysis also illustrated that the patients in the high RS group present high mortality according to the CGGA dataset , as shown in **Figure 3H**. Taken together, these results suggest the feasibility and validity of our RS formula.

### Independent prognostic value of the necroptosis-related long non-coding RNA signature

**Figure 4:**
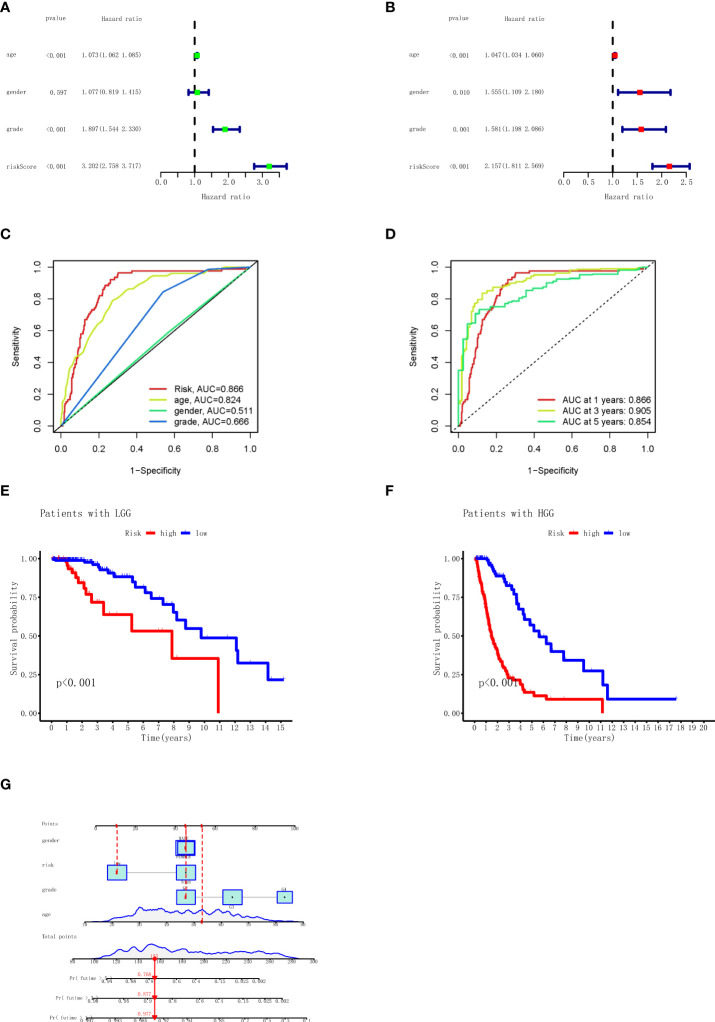
Univariate and multivariate Cox regression analyses, Kaplan–Meier survival analysis, and prognostic nomogram model for the risk score. **(A)** Univariate Cox regression analysis for TCGA (grade: the degree of glioma differentiation, G2 to G4). **(B)** Multivariate Cox regression analyses for TCGA. **(C)** AUC in ROC analysis for risk signature age, gender, grade, and risk score in TCGA. **(D)** AUC in ROC analysis for risk signature at 1-, 3-, and 5-year survival time in TCGA. **(E)** Kaplan–Meier survival analysis of the LGG high‐risk and low‐risk groups based on the nine necroptosis-related lncRNAs. **(F)** Kaplan–Meier survival analysis of the HGG high‐risk and low‐risk groups based on the nine necroptosis-related lncRNAs. **(G)** The nomogram prediction for 1-, 3-, and 5-year overall survival probability for glioma patients. TCGA, The Cancer Genome Atlas; AUC, area under the receiver operating characteristic curve; ROC, receiver operating characteristic; LGG, low-grade glioma; HGG, high-grade glioma.

To validate the predictive value of RS in patients with glioma, we used univariate Cox and multivariate Cox regression analyses to show whether there was a difference in outcomes between the high-risk and low-risk groups. As shown in **Figures 4A, B**, the results show significant differences in RS between the two groups (univariate Cox, hazard ratio (HR) = 3.202, 95% confidence interval (CI) = 2.758–3.717, p < 0.001; multivariate Cox, HR = 2.157, 95% CI = 1.811–2.569, p < 0.001). To assess the predictive specificity and sensitivity of the clinical features and RS formula for survival prognosis in patients with glioma, we calculated the area under the ROC curve (AUC) of the RS, as shown in **Figure 4C**. The AUC of the risk score was 0.886. The specificity and sensitivity of 1-, 3-, and 5-year prognoses also used the ROC curve, as shown in **Figure 4D**. The AUC values of 1, 3, and 5 years were 0.866, 0.905, and 0.854, respectively, suggesting that there were strong correlations between RS and OS. We also analyzed the OS of LGG and HGG in TCGA database using the RS formula, and the results showed that LGG and HGG patients with high RS had a shorter survival time, as shown in **Figures 4E, F**. To demonstrate in more detail the predictability at different grades, we analyzed the OS of the independent prognostic value of the necroptosis-related lncRNA signature among different grades of gliomas (see **Supplementary Figure 2**). These results demonstrated that the prognostic RS model of the nine necroptosis-related lncRNA for glioma is considerably reliable. Furthermore, the clinical features and RS were incorporated to establish a nomogram model for predicting the rates of OS at the 1, 3, and 5 years in glioma; the score scale at the top of the nomogram was used to measure clinical features and RS, and then the measured scores were added to estimate the probability of survival in glioma patients for 1, 3, and 5 years, as shown in **Figure 4G**.

### Functional analyses of necroptosis-related long non-coding RNAs

**Figure 5:**
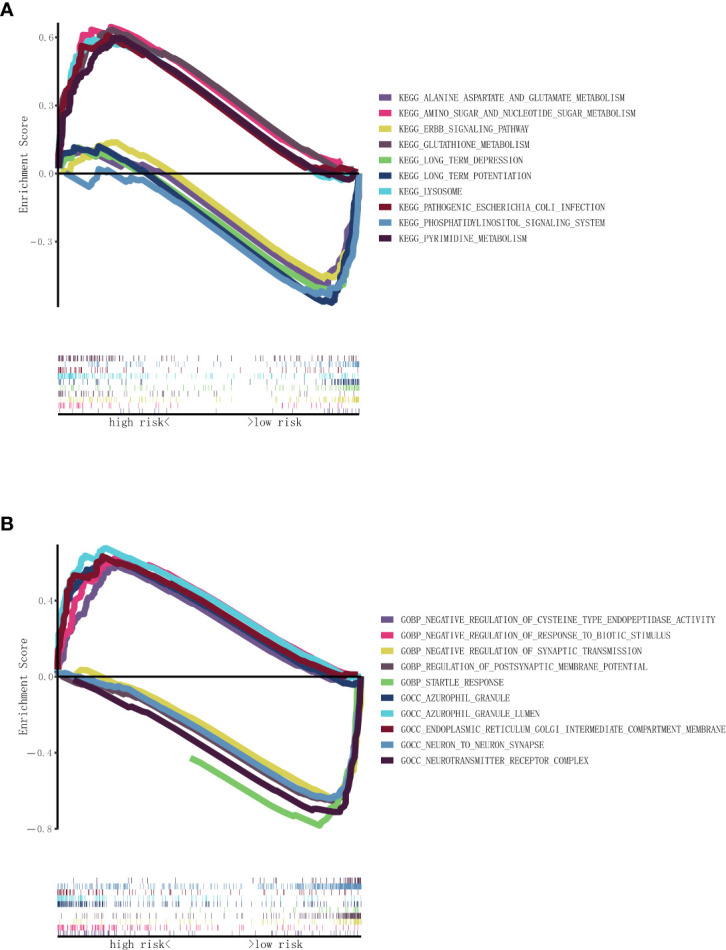
Functional enrichment analysis based on nine necroptosis-related lncRNAs between the two risk groups in TCGA. **(A)** KEGG analysis of nine necroptosis-related lncRNAs. **(B)** GO analysis of nine necroptosis-related lncRNAs. lncRNAs, long non-coding RNAs; TCGA, The Cancer Genome Atlas; KEGG, Kyoto Encyclopedia of Genes and Genomes; GO, Gene Ontology.

GSEA was conducted to investigate the KEGG and GO related to the necroptosis-related lncRNA signature. The results of the KEGG demonstrated that primary increased functions in the high RS group were enriched in pathogenic *Escherichia coli* infection, pyrimidine metabolism, and glutathione metabolism and that primary decreased functions in the high RS group were enriched in long-term depression, long-term potentiation, and phosphatidylinositol signaling system, as shown in **Figure 5A**. The results of the GO demonstrated that primary increased functions in the high RS group were enriched in azurophil granule lumen, negative regulation of cysteine-type endopeptidase activity, and endoplasmic reticulum Golgi intermediate compartment membrane and that primary decreased functions in the high RS group were enriched in negative regulation of synaptic transmission, neuron-to-neuron synapse, and startle response, as shown in **Figure 5B**.

### Immune cell infiltration subtypes and immune functions

**Figure 6:**
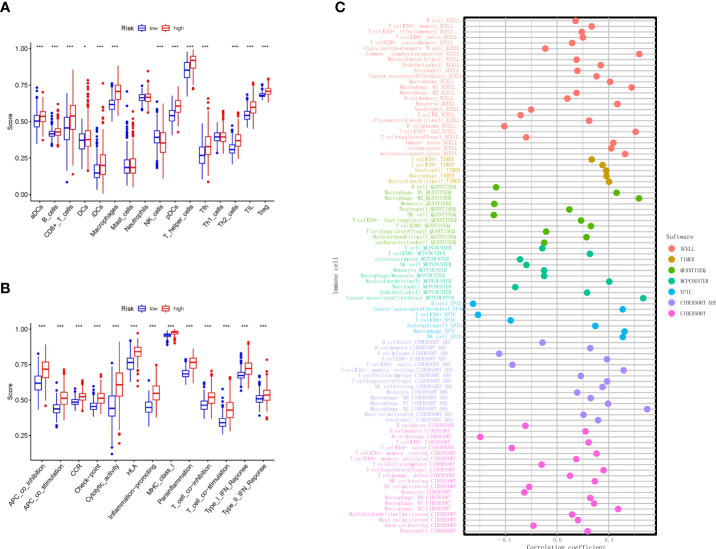
Immune algorithms employed in comparison based on nine necroptosis-related lncRNAs between the two risk groups in TCGA. **(A)** Comparison of the ssGSEA scores of nine necroptosis-related lncRNAs for immune cells in TCGA. **(B)** Comparison of the ssGSEA scores of nine necroptosis-related lncRNAs for immune-related pathways in TCGA. **(C)** Bubble graph for the infiltration levels of immune cells under XCELL, TIMER, QUANTISEQ, MCPCOUNTER, EPIC, CIBERSOFT ABS, and CIBERSOFT algorithms. **p < 0.01, and ***p < 0.001. lncRNAs, long non-coding RNAs; TCGA, The Cancer Genome Atlas; ssGSEA, single-sample Gene Set Enrichment Analysis.

To understand the differences in immune cell subset expression in the glioma immune microenvironment, we calculated and compared the expression of different infiltrating immune cells, as shown in **Figure 6A**; Activated dendritic cells (aDCs), B cells, CD8+ T cells, DCs, Macrophages, NK cells, Plasmacytoid DCs (pDCs), T helper cells, Tfh, Th2 cells, TILs, and Tregs were significantly upregulated in the high RS group. In addition, ssGSEA was performed to assess the level of enrichment of immune functions, as shown in **Figure 6B**; immune functions in Antigen-presenting cell (APC) co-inhibition, APC co-stimulation, CC chemokine receptor (CCR), Check-point, Cytolytic activity, human leukocyte antigen (HLA), Inflammation-promoting, MHC class I, Parainflammation, T-cell co-inhibition, T-cell co-stimulation, Type I IFN response, and Type II IFN response were significantly enriched in the high RS group. To further investigate the role of infiltrating immune cells in the glioma immune microenvironment, we calculated and compared the correlation of common infiltrating immune cells in two groups through multiple databases. The most commonly used CIBERSOFT (**Figure 6C**) revealed that B cell memory, T cell CD8+, T cell CD4+ memory resting, T cell CD4+ memory activated, T cell regulatory (Tregs), T cell gamma delta, NK cell resting, Macrophage M0, Macrophage M1, Macrophage M2, Myeloid dendritic cell activated, Mast cell activated, and Neutrophil were positively correlated with RS and that B cell naive, B cell plasma, T cell CD4+ naive, T cell follicular helper, NK cell activated, Monocyte, and Mast cell resting were negatively correlated with RS.

### The characteristics of tumor immune microenvironment and immune checkpoints

**Figure 7:**
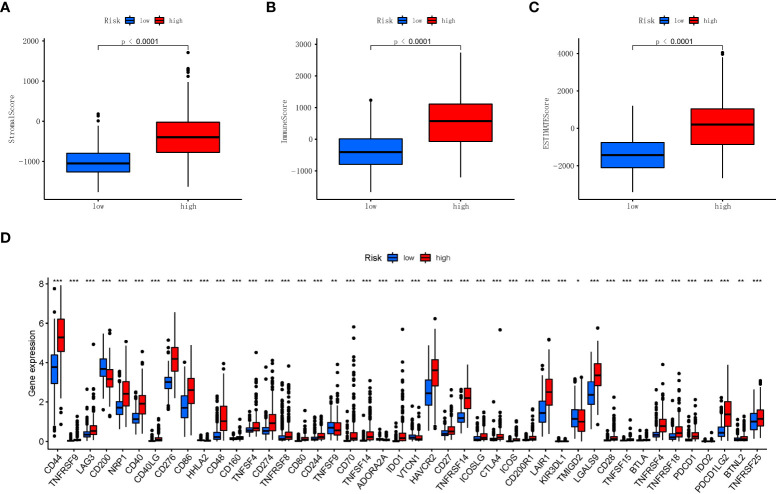
Association of stromal, immune scores, and immune checkpoint with two risk groups in TCGA. **(A)** Comparisons of stromal score between the two risk groups in TCGA. **(B)** Comparisons of immune scores between the two risk groups in TCGA. **(C)** Comparisons of ESTIMATE score between the two risk groups in TCGA. **(D)** Boxplot of the expression levels of immune checkpoint between the two risk groups in TCGA. *p < 0.05, **p < 0.01, ***p < 0.001. TCGA, The Cancer Genome Atlas.

Previous studies have shown that the tumor microenvironment is composed of tumor cells, stromal cells, and immune cells. Through the ‘ESTIMATE’ R software package, we used the tumor microenvironment score to measure the difference in the level of infiltration of stromal and immune cells between the low RS and high RS groups, and the results showed that the high RS groups had higher stromal (**Figure 7A**), immune (**Figure 7B**), and ESTIMATE scores (**Figure 7C**). Next, in order to study the potential changes of immune checkpoints between the high and low RS groups, we compared the expression levels of common immune checkpoints related lncRNAs in the two groups, as shown in **Figure 7D**, among which the lncRNAs with the most significant differences were CD44, CD276, CD86, CD48, and HAVCR2, etc. Furthermore, the association of our established RS with neurotransmitters such as GABA is also presented in **Supplementary Figure 1**.

## Discussion

Glioma, the most common brain tumor, remains a great threat to human health throughout the world. Therefore, identification of the prognosis of glioma patients and gene signatures can improve the prognosis of patients and treatment of life; studies have shown that lncRNA plays an important role in predicting the prognosis of glioma, but there are no studies on necroptosis-related lncRNAs in predicting the prognosis of glioma so far. Herein, we constructed the necroptosis-related lncRNAs model from the RNA-seq in TCGA database and explored its effect on the prognosis of glioma.

In this study, 173 differentially expressed necroptosis-related lncRNAs were screened. Finally, after Cox regression and LASSO analysis, a total of nine lncRNAs were incorporated into the RS formula to predict the OS of glioma patients. Among those lncRNAs, MIR22HG shows that glioma progression can be inhibited by downregulation of micNA-9/CPEB3 (20) and inhibits glioblastoma progression through Wnt/beta-catenin signaling (21). PAXIP1.AS2 has been reported as ferroptosis-associated lncRNA that could affect the radiotherapy response for glioma (22). CRNDE has been reported to promote the proliferation, migration, and invasion of glioma through attenuating miR-384/PIWIL4/STAT3 axis (23) and facilitating EGFR activation to modulate glioma growth (24). Moreover, the knockdown of CRNDE could increase the sensitivity of temozolomide chemotherapy for glioma and modulate the prognosis of patients (25). PCED1B.AS1 has been reported to cooperate with Mir-194-5p to promote the proliferation of glioma and inhibit the apoptosis of glioma cells (26) and promote glioblastoma genesis through upregulation of HIF-1alpha (27). LBX2.AS1 has been reported to sponge miR-491-5p to further upregulate LIF and modulate the progression of glioma (28). It was also found *in vitro* that lBX2.AS1 silencing could activate Akt/GSK3β pathway to inhibit the proliferation and metabolism of glioma cells (29). It has been reported that overexpression of LINC00641 in glioma can inhibit cell proliferation and promote cell apoptosis and achieved through LINC00641/Mir-4262/NRGN axis (30). Although AC083799.1, C10orf55, and GNas.AS1 have not been reported in glioma, some studies have found that they play important roles in the occurrence and development of other tumors, such as breast cancer (31), colon cancer (32), acute myeloid leukemia (33), and endometrial cancer (34).

In order to further investigate the relationship between necroptosis-related lncRNAs and glioma, GSEA that showed pathogenic *Escherichia coli* infection, pyrimidine metabolism, and glutathione metabolism were enriched in the high RS group, while long-term depression, long-term potentiation, and phosphatidylinositol signaling system were enriched in the low RS group. A study suggests that targeting pyrimidine metabolism reprogramming in cancer stem cells and lower expression of pyrimidine metabolism indicates a better prognosis of glioblastoma (35), and pharmacologic inhibition of glutathione metabolism could be valuable for patients with IDH1-mutated glioma (36).

Moreover, our results show that more NK cells were infiltrated in the glioma immune microenvironment of the low-risk group, suggesting a correlation between NK cells and glioma necroptosis. Subsequent analyses of the correlation of infiltrating glioma immune cells in multiple databases have shown a negative correlation between NK cells and necroptosis of glioma, suggesting that NK cells may be an effective target for glioma immunotherapy. Despite the fact that central nervous system (CNS) was considered immunological isolation, growing evidence showed that immune cells including infiltrated macrophages, lymphocytes, DCs, and NK cells play crucial roles in cancer progression (37). Therefore, accumulating methods of immunotherapy were introduced in glioma therapy such as DC vaccines, CAR-T, or immune checkpoint blockade (38). Although the majority of current clinical trials did not exhibit outstanding improvement, numerous clinical trials indeed proved that these methods are promising for some patients. Indeed, the previous study demonstrates the factors that influence the outcome of immunotherapy against the tumor, which concludes the evidence of the importance of sex, race, etc. (39). Our study, however, provides the gene indicators of glioma patients based on transcriptomic data, further improving the evaluation of patients’ prognosis. In addition, much evidence has shown that lncRNAs implicate the tumor microenvironment (40, 41).

Immune escape from necroptosis and inflammation play a vital role in cancer (42); the abnormality of the immune checkpoint will make the immune cells unable to produce an effective anti-tumor immune response, and the tumor will form immune escape (43). Therefore, we analyzed the gliomas’ immune checkpoint, and multiple abnormal immune checkpoints were found in the high RS group, and many of them have been reported to participate in the regulation of the growth of gliomas, such as CD44, CD86, and HAVCR2 (44–46).

Current studies have largely proved that lncRNA can interact with microRNA (miRNA) by the so-called ‘sponging’-like mechanisms because of specific binding sites. There are very limited data describing the nine necroptosis-related lncRNAs (MIR22HG, AC083799.1, PAXIP1.AS2, C10orf55, GNAS.AS1, CRNDE, PCED1B.AS1, LBX2.AS1, and LINC00641). Hence, we constructed the potential regulated miRNA network by miRcode online databases (http://mircode.org/), and the potential interacted miRNAs are listed in **Supplementary Table 3**, However, we observed that only MIR22HG, AC083799.1, C10orf55, GNAS-AS1, CRNDE, LBX2-AS1, and LINC00641 have miRNA binding sites.

Recently, the prognostic RS models of necroptosis-related lncRNAs were constructed in many tumors including colon cancer (47), stomach adenocarcinoma (48), gastric cancer (49), and breast cancer (50). However, the role of necroptosis-related lncRNAs in the prognosis of glioma remains unclear; we therefore conducted this research and established a nine-necroptosis-related lncRNA prognostic RS model, which might be essential for the future development of individual effective therapies in glioma.

## Conclusion

A nine-necroptosis-related lncRNA RS formula was constructed that can effectively predict the prognosis of glioma patients and provided a theoretical basis and the potential therapeutic targets for necroptosis-related lncRNAs in immunotherapy for gliomas.

## Data availability statement

The datasets presented in this study can be found in online repositories. The names of the repository/repositories and accession number(s) can be found in the article/**Supplementary Material**.

## Author contributions

All authors listed have made a substantial, direct, and intellectual contribution to the work and approved it for publication.

## Conflict of interest

The authors declare that the research was conducted in the absence of any commercial or financial relationships that could be construed as a potential conflict of interest.

## Publisher’s note

All claims expressed in this article are solely those of the authors and do not necessarily represent those of their affiliated organizations, or those of the publisher, the editors and the reviewers. Any product that may be evaluated in this article, or claim that may be made by its manufacturer, is not guaranteed or endorsed by the publisher.

## Supplementary material

Supplementary Figure 1 Differential expression of GABA receptors in high and low RS cohort.

Supplementary Figure 2 Kaplan–Meier survival analysis of the different grades high‐risk and low‐risk groups based on the 9 necroptosis-related LncRNAs.

Supplementary Table 2 The necroptosis process related lncRNA signatures.

Supplementary Table 3 Predicted binding miRNAs of selected lncRNAs.
