# Supplementary material for: A novel signature based on necroptosis-related long non-coding RNAs for predicting prognosis of patients with glioma
Source: Front Oncol. 2022 Aug 10;12:940220. doi: 10.3389/fonc.2022.940220 (PMC9399791; doi:10.3389/fonc.2022.940220)
Supplement: Supplementary file 1 [file Table_1.docx]

**Supplementary Table 1. necroptosis-related genes and source**

| **Genes** | **Source** |
| --- | --- |

AIM2 Periasamy S, Le HT, Duffy EB, Chin H, Harton JA. Inflammasome-Independent NLRP3 Restriction of a Protective Early Neutrophil Response to Pulmonary Tularemia. PLoS Pathog. 2016 Dec 7;12(12):e1006059. doi: 10.1371/journal.ppat.1006059. PMID: 27926940; PMCID: PMC5142794.

BAK1 Karch J, Kanisicak O, Brody MJ, Sargent MA, Michael DM, Molkentin JD. Necroptosis Interfaces with MOMP and the MPTP in Mediating Cell Death. PLoS One. 2015 Jun 10;10(6):e0130520. doi: 10.1371/journal.pone.0130520. PMID: 26061004; PMCID: PMC4465034.

BAX Sundarraj K, Raghunath A, Panneerselvam L, Perumal E. Fisetin, a phytopolyphenol, targets apoptotic and necroptotic cell death in HepG2 cells. Biofactors. 2020 Jan;46(1):118-135. doi: 10.1002/biof.1577. Epub 2019 Oct 21. PMID: 31634424.

CASP1 Kitur K, Wachtel S, Brown A, Wickersham M, Paulino F, Peñaloza HF, Soong G, Bueno S, Parker D, Prince A. Necroptosis Promotes Staphylococcus aureus Clearance by Inhibiting Excessive Inflammatory Signaling. Cell Rep. 2016 Aug 23;16(8):2219-2230. doi: 10.1016/j.celrep.2016.07.039. Epub 2016 Aug 11. PMID: 27524612; PMCID: PMC5001919.

CASP3 Dojo Soeandy C, Elia AJ, Cao Y, Rodgers C, Huang S, Elia AC, Henderson JT. Necroptotic-Apoptotic Regulation in an Endothelin-1 Model of Cerebral Ischemia. Cell Mol Neurobiol. 2021 Nov;41(8):1727-1742. doi: 10.1007/s10571-020-00942-y. Epub 2020 Aug 25. PMID: 32844322.

CASP4 Dojo Soeandy C, Elia AJ, Cao Y, Rodgers C, Huang S, Elia AC, Henderson JT. Necroptotic-Apoptotic Regulation in an Endothelin-1 Model of Cerebral Ischemia. Cell Mol Neurobiol. 2021 Nov;41(8):1727-1742. doi: 10.1007/s10571-020-00942-y. Epub 2020 Aug 25. PMID: 32844322.

CASP5 Mandal P, Feng Y, Lyons JD, Berger SB, Otani S, DeLaney A, Tharp GK, Maner-Smith K, Burd EM, Schaeffer M, Hoffman S, Capriotti C, Roback L, Young CB, Liang Z, Ortlund EA, DiPaolo NC, Bosinger S, Bertin J, Gough PJ, Brodsky IE, Coopersmith CM, Shayakhmetov DM, Mocarski ES. Caspase-8 Collaborates with Caspase-11 to Drive Tissue Damage and Execution of Endotoxic Shock. Immunity. 2018 Jul 17;49(1):42-55.e6. doi: 10.1016/j.immuni.2018.06.011. PMID: 30021146; PMCID: PMC6064639.

CASP6 van Raam BJ, Ehrnhoefer DE, Hayden MR, Salvesen GS. Intrinsic cleavage of receptor-interacting protein kinase-1 by caspase-6. Cell Death Differ. 2013 Jan;20(1):86-96. doi: 10.1038/cdd.2012.98. Epub 2012 Aug 3. PMID: 22858542; PMCID: PMC3524638.

CASP8 Newton K, Wickliffe KE, Dugger DL, Maltzman A, Roose-Girma M, Dohse M, Kőműves L, Webster JD, Dixit VM. Cleavage of RIPK1 by caspase-8 is crucial for limiting apoptosis and necroptosis. Nature. 2019 Oct;574(7778):428-431. doi: 10.1038/s41586-019-1548-x. Epub 2019 Sep 11. PMID: 31511692.

CASP9 Usategui-Martín R, Puertas-Neyra K, Galindo-Cabello N, Hernández-Rodríguez LA, González-Pérez F, Rodríguez-Cabello JC, González-Sarmiento R, Pastor JC, Fernandez-Bueno I. Retinal Neuroprotective Effect of Mesenchymal Stem Cells Secretome Through Modulation of Oxidative Stress, Autophagy, and Programmed Cell Death. Invest Ophthalmol Vis Sci. 2022 Apr 1;63(4):27. doi: 10.1167/iovs.63.4.27. PMID: 35486068; PMCID: PMC9055551.

CHMP2A Hattori T, Takahashi Y, Chen L, Tang Z, Wills CA, Liang X, Wang HG. Targeting the ESCRT-III component CHMP2A for noncanonical Caspase-8 activation on autophagosomal membranes. Cell Death Differ. 2021 Feb;28(2):657-670. doi: 10.1038/s41418-020-00610-0. Epub 2020 Aug 17. PMID: 32807832; PMCID: PMC7862398.

CHMP2B Gong YN, Guy C, Olauson H, Becker JU, Yang M, Fitzgerald P, Linkermann A, Green DR. ESCRT-III Acts Downstream of MLKL to Regulate Necroptotic Cell Death and Its Consequences. Cell. 2017 Apr 6;169(2):286-300.e16. doi: 10.1016/j.cell.2017.03.020. PMID: 28388412; PMCID: PMC5443414.

CHMP3 Gong YN, Guy C, Olauson H, Becker JU, Yang M, Fitzgerald P, Linkermann A, Green DR. ESCRT-III Acts Downstream of MLKL to Regulate Necroptotic Cell Death and Its Consequences. Cell. 2017 Apr 6;169(2):286-300.e16. doi: 10.1016/j.cell.2017.03.020. PMID: 28388412; PMCID: PMC5443414.

CHMP4A Gong YN, Guy C, Olauson H, Becker JU, Yang M, Fitzgerald P, Linkermann A, Green DR. ESCRT-III Acts Downstream of MLKL to Regulate Necroptotic Cell Death and Its Consequences. Cell. 2017 Apr 6;169(2):286-300.e16. doi: 10.1016/j.cell.2017.03.020. PMID: 28388412; PMCID: PMC5443414.

CHMP4B Gong YN, Guy C, Olauson H, Becker JU, Yang M, Fitzgerald P, Linkermann A, Green DR. ESCRT-III Acts Downstream of MLKL to Regulate Necroptotic Cell Death and Its Consequences. Cell. 2017 Apr 6;169(2):286-300.e16. doi: 10.1016/j.cell.2017.03.020. PMID: 28388412; PMCID: PMC5443414.

CHMP4C Gong YN, Guy C, Olauson H, Becker JU, Yang M, Fitzgerald P, Linkermann A, Green DR. ESCRT-III Acts Downstream of MLKL to Regulate Necroptotic Cell Death and Its Consequences. Cell. 2017 Apr 6;169(2):286-300.e16. doi: 10.1016/j.cell.2017.03.020. PMID: 28388412; PMCID: PMC5443414.

CHMP6 Gong YN, Guy C, Olauson H, Becker JU, Yang M, Fitzgerald P, Linkermann A, Green DR. ESCRT-III Acts Downstream of MLKL to Regulate Necroptotic Cell Death and Its Consequences. Cell. 2017 Apr 6;169(2):286-300.e16. doi: 10.1016/j.cell.2017.03.020. PMID: 28388412; PMCID: PMC5443414.

CHMP7 Gong YN, Guy C, Olauson H, Becker JU, Yang M, Fitzgerald P, Linkermann A, Green DR. ESCRT-III Acts Downstream of MLKL to Regulate Necroptotic Cell Death and Its Consequences. Cell. 2017 Apr 6;169(2):286-300.e16. doi: 10.1016/j.cell.2017.03.020. PMID: 28388412; PMCID: PMC5443414.

CYCS Bao C, Sun Y, Dwarakanath B, Dong Y, Huang Y, Wu X, Guha C, Kong L, Lu JJ. Carbon ion triggered immunogenic necroptosis of nasopharyngeal carcinoma cells involving necroptotic inhibitor BCL-x. J Cancer. 2021 Jan 1;12(5):1520-1530. doi: 10.7150/jca.46316. PMID: 33531997; PMCID: PMC7847655.

ELANE Galluzzi, L., Vitale, I., Aaronson, S. A., Abrams, J. M., Adam, D., Agostinis, P., Alnemri, E. S., Altucci, L., Amelio, I., Andrews, D. W., Annicchiarico-Petruzzelli, M., Antonov, A. V., Arama, E., Baehrecke, E. H., Barlev, N. A., Bazan, N. G., Bernassola, F., Bertrand, M., Bianchi, K., Blagosklonny, M. V., … Kroemer, G. (2018). Molecular mechanisms of cell death: recommendations of the Nomenclature Committee on Cell Death 2018. Cell death and differentiation, 25(3), 486–541. https://doi.org/10.1038/s41418-017-0012-4

GPX4 Basit F, van Oppen LM, Schöckel L, Bossenbroek HM, van Emst-de Vries SE, Hermeling JC, Grefte S, Kopitz C, Heroult M, Hgm Willems P, Koopman WJ. Mitochondrial complex I inhibition triggers a mitophagy-dependent ROS increase leading to necroptosis and ferroptosis in melanoma cells. Cell Death Dis. 2017 Mar 30;8(3):e2716. doi: 10.1038/cddis.2017.133. PMID: 28358377; PMCID: PMC5386536.

GSDMA Frank D, Vince JE. Pyroptosis versus necroptosis: similarities, differences, and crosstalk. Cell Death Differ. 2019 Jan;26(1):99-114. doi: 10.1038/s41418-018-0212-6. Epub 2018 Oct 19. PMID: 30341423; PMCID: PMC6294779.

GSDMB Frank D, Vince JE. Pyroptosis versus necroptosis: similarities, differences, and crosstalk. Cell Death Differ. 2019 Jan;26(1):99-114. doi: 10.1038/s41418-018-0212-6. Epub 2018 Oct 19. PMID: 30341423; PMCID: PMC6294779.

GSDMC Frank D, Vince JE. Pyroptosis versus necroptosis: similarities, differences, and crosstalk. Cell Death Differ. 2019 Jan;26(1):99-114. doi: 10.1038/s41418-018-0212-6. Epub 2018 Oct 19. PMID: 30341423; PMCID: PMC6294779.

GSDMD Frank D, Vince JE. Pyroptosis versus necroptosis: similarities, differences, and crosstalk. Cell Death Differ. 2019 Jan;26(1):99-114. doi: 10.1038/s41418-018-0212-6. Epub 2018 Oct 19. PMID: 30341423; PMCID: PMC6294779.

GSDME Frank D, Vince JE. Pyroptosis versus necroptosis: similarities, differences, and crosstalk. Cell Death Differ. 2019 Jan;26(1):99-114. doi: 10.1038/s41418-018-0212-6. Epub 2018 Oct 19. PMID: 30341423; PMCID: PMC6294779.

GZMA Lee PY, Park BC, Chi SW, Bae KH, Kim S, Cho S, Kang S, Kim JH, Park SG. Histone H4 is cleaved by granzyme A during staurosporine-induced cell death in B-lymphoid Raji cells. BMB Rep. 2016 Oct;49(10):560-565. doi: 10.5483/bmbrep.2016.49.10.105. PMID: 27439606; PMCID: PMC5227298.

GZMB Snyder AG, Hubbard NW, Messmer MN, Kofman SB, Hagan CE, Orozco SL, Chiang K, Daniels BP, Baker D, Oberst A. Intratumoral activation of the necroptotic pathway components RIPK1 and RIPK3 potentiates antitumor immunity. Sci Immunol. 2019 Jun 21;4(36):eaaw2004. doi: 10.1126/sciimmunol.aaw2004. PMID: 31227597; PMCID: PMC6831211.

HMGB1 Fan H, Tang HB, Chen Z, Wang HQ, Zhang L, Jiang Y, Li T, Yang CF, Wang XY, Li X, Wu SX, Zhang GL. Inhibiting HMGB1-RAGE axis prevents pro-inflammatory macrophages/microglia polarization and affords neuroprotection after spinal cord injury. J Neuroinflammation. 2020 Oct 9;17(1):295. doi: 10.1186/s12974-020-01973-4. PMID: 33036632; PMCID: PMC7547440.

IL18 Yang J, Zhao Y, Zhang L, Fan H, Qi C, Zhang K, Liu X, Fei L, Chen S, Wang M, Kuang F, Wang Y, Wu S. RIPK3/MLKL-Mediated Neuronal Necroptosis Modulates the M1/M2 Polarization of Microglia/Macrophages in the Ischemic Cortex. Cereb Cortex. 2018 Jul 1;28(7):2622-2635. doi: 10.1093/cercor/bhy089. PMID: 29746630; PMCID: PMC5998990.

IL1A He C, Liu Y, Huang Z, Yang Z, Zhou T, Liu S, Hao Z, Wang J, Feng Q, Liu Y, Cao Y, Liu X. A specific RIP3+ subpopulation of microglia promotes retinopathy through a hypoxia-triggered necroptotic mechanism. Proc Natl Acad Sci U S A. 2021 Mar 16;118(11):e2023290118. doi: 10.1073/pnas.2023290118. PMID: 33836603; PMCID: PMC7980367.

IL1B Idrovo JP, Boe DM, Kaahui S, Yang WL, Kovacs EJ. Hepatic inflammation after burn injury is associated with necroptotic cell death signaling. J Trauma Acute Care Surg. 2020 Oct;89(4):768-774. doi: 10.1097/TA.0000000000002865. PMID: 33017135; PMCID: PMC8386183.

IL6 Riegler AN, Brissac T, Gonzalez-Juarbe N, Orihuela CJ. Necroptotic Cell Death Promotes Adaptive Immunity Against Colonizing Pneumococci. Front Immunol. 2019 Apr 4;10:615. doi: 10.3389/fimmu.2019.00615. PMID: 31019504; PMCID: PMC6459137.

IRF1 Xiong Y, Li L, Zhang L, Cui Y, Wu C, Li H, Chen K, Yang Q, Xiang R, Hu Y, Huang S, Wei Y, Yang S. The bromodomain protein BRD4 positively regulates necroptosis via modulating MLKL expression. Cell Death Differ. 2019 Oct;26(10):1929-1941. doi: 10.1038/s41418-018-0262-9. Epub 2019 Jan 15. PMID: 30644439; PMCID: PMC6748082.

IRF2 Rosenbaum SR, Wilski NA, Aplin AE. Fueling the Fire: Inflammatory Forms of Cell Death and Implications for Cancer Immunotherapy. Cancer Discov. 2021 Feb;11(2):266-281. doi: 10.1158/2159-8290.CD-20-0805. Epub 2021 Jan 15. PMID: 33451983; PMCID: PMC7858229.

NLRC4 Paudel S, Ghimire L, Jin L, Baral P, Cai S, Jeyaseelan S. NLRC4 suppresses IL-17A-mediated neutrophil-dependent host defense through upregulation of IL-18 and induction of necroptosis during Gram-positive pneumonia. Mucosal Immunol. 2019 Jan;12(1):247-257. doi: 10.1038/s41385-018-0088-2. Epub 2018 Oct 2. PMID: 30279514; PMCID: PMC6301100.

NLRP1 Atkin-Smith GK. Phagocytic clearance of apoptotic, necrotic, necroptotic and pyroptotic cells. Biochem Soc Trans. 2021 Apr 30;49(2):793-804. doi: 10.1042/BST20200696. PMID: 33843978; PMCID: PMC8106503.

NLRP2 Favor OK, Pestka JJ, Bates MA, Lee KSS. Centrality of Myeloid-Lineage Phagocytes in Particle-Triggered Inflammation and Autoimmunity. Front Toxicol. 2021 Nov 4;3:777768. doi: 10.3389/ftox.2021.777768. PMID: 35295146; PMCID: PMC8915915.

NLRP3 Conos SA, Chen KW, De Nardo D, Hara H, Whitehead L, Núñez G, Masters SL, Murphy JM, Schroder K, Vaux DL, Lawlor KE, Lindqvist LM, Vince JE. Active MLKL triggers the NLRP3 inflammasome in a cell-intrinsic manner. Proc Natl Acad Sci U S A. 2017 Feb 7;114(6):E961-E969. doi: 10.1073/pnas.1613305114. Epub 2017 Jan 17. Erratum in: Proc Natl Acad Sci U S A. 2017 Jul 11;114(28):E5762-E5763. PMID: 28096356; PMCID: PMC5307433.

NLRP6 Ghimire L, Paudel S, Jin L, Baral P, Cai S, Jeyaseelan S. NLRP6 negatively regulates pulmonary host defense in Gram-positive bacterial infection through modulating neutrophil recruitment and function. PLoS Pathog. 2018 Sep 24;14(9):e1007308. doi: 10.1371/journal.ppat.1007308. PMID: 30248149; PMCID: PMC6171945.

NLRP7 Missiakas D, Winstel V. Selective Host Cell Death by *Staphylococcus aureus*: A Strategy for Bacterial Persistence. Front Immunol. 2021 Jan 21;11:621733. doi: 10.3389/fimmu.2020.621733. PMID: 33552085; PMCID: PMC7859115.

NOD1 Zhang L, Wang X, Chen S, Wang S, Tu Z, Zhang G, Zhu H, Li X, Xiong J, Liu Y. Medium-Chain Triglycerides Attenuate Liver Injury in Lipopolysaccharide-Challenged Pigs by Inhibiting Necroptotic and Inflammatory Signaling Pathways. Int J Mol Sci. 2018 Nov 21;19(11):3697. doi: 10.3390/ijms19113697. PMID: 30469452; PMCID: PMC6274951.

NOD2 Zhu H, Wang H, Wang S, Tu Z, Zhang L, Wang X, Hou Y, Wang C, Chen J, Liu Y. Flaxseed Oil Attenuates Intestinal Damage and Inflammation by Regulating Necroptosis and TLR4/NOD Signaling Pathways Following Lipopolysaccharide Challenge in a Piglet Model. Mol Nutr Food Res. 2018 May;62(9):e1700814. doi: 10.1002/mnfr.201700814. Epub 2018 Apr 14. PMID: 29510469.

PJVK Demarco B, Danielli S, Fischer FA, Bezbradica JS. How Pyroptosis Contributes to Inflammation and Fibroblast-Macrophage Cross-Talk in Rheumatoid Arthritis. Cells. 2022 Apr 12;11(8):1307. doi: 10.3390/cells11081307. PMID: 35455985; PMCID: PMC9028325.

PLCG1 Tang D, Kang R, Berghe TV, Vandenabeele P, Kroemer G. The molecular machinery of regulated cell death. Cell Res. 2019 May;29(5):347-364. doi: 10.1038/s41422-019-0164-5. Epub 2019 Apr 4. PMID: 30948788; PMCID: PMC6796845.

PRKACA Holczbauer Á, Wangensteen KJ, Shin S. Cellular origins of regenerating liver and hepatocellular carcinoma. JHEP Rep. 2021 Dec 13;4(4):100416. doi: 10.1016/j.jhepr.2021.100416. PMID: 35243280; PMCID: PMC8873941.

PYCARD Lim J, Park H, Heisler J, Maculins T, Roose-Girma M, Xu M, Mckenzie B, van Lookeren Campagne M, Newton K, Murthy A. Autophagy regulates inflammatory programmed cell death via turnover of RHIM-domain proteins. Elife. 2019 Jul 9;8:e44452. doi: 10.7554/eLife.44452. PMID: 31287416; PMCID: PMC6615860.

SCAF11 Li H, Li T, Zhang X. Identification of a Pyroptosis-Related Prognostic Signature Combined With Experiments in Hepatocellular Carcinoma. Front Mol Biosci. 2022 Mar 4;9:822503. doi: 10.3389/fmolb.2022.822503. PMID: 35309514; PMCID: PMC8931679.

TIRAP Legarda D, Justus SJ, Ang RL, Rikhi N, Li W, Moran TM, Zhang J, Mizoguchi E, Zelic M, Kelliher MA, Blander JM, Ting AT. CYLD Proteolysis Protects Macrophages from TNF-Mediated Auto-necroptosis Induced by LPS and Licensed by Type I IFN. Cell Rep. 2016 Jun 14;15(11):2449-61. doi: 10.1016/j.celrep.2016.05.032. Epub 2016 Jun 2. PMID: 27264187; PMCID: PMC4909532.

TNF Chen AQ, Fang Z, Chen XL, Yang S, Zhou YF, Mao L, Xia YP, Jin HJ, Li YN, You MF, Wang XX, Lei H, He QW, Hu B. Microglia-derived TNF-α mediates endothelial necroptosis aggravating blood brain-barrier disruption after ischemic stroke. Cell Death Dis. 2019 Jun 20;10(7):487. doi: 10.1038/s41419-019-1716-9. PMID: 31221990; PMCID: PMC6586814.

TP53 Bebber CM, Thomas ES, Stroh J, Chen Z, Androulidaki A, Schmitt A, Höhne MN, Stüker L, de Pádua Alves C, Khonsari A, Dammert MA, Parmaksiz F, Tumbrink HL, Beleggia F, Sos ML, Riemer J, George J, Brodesser S, Thomas RK, Reinhardt HC, von Karstedt S. Ferroptosis response segregates small cell lung cancer (SCLC) neuroendocrine subtypes. Nat Commun. 2021 Apr 6;12(1):2048. doi: 10.1038/s41467-021-22336-4. PMID: 33824345; PMCID: PMC8024350.

TP63 Wang Y, Lu H, Wang Z, Li Y, Chen X. TGF-β1 Promotes Autophagy and Inhibits Apoptosis in Breast Cancer by Targeting TP63. Front Oncol. 2022 Apr 11;12:865067. doi: 10.3389/fonc.2022.865067. PMID: 35480110; PMCID: PMC9035888.

|  |  |
| --- | --- |
